# Development of a core outcome set for clinical trials targeting interventions aiming to improve adherence to appropriate polypharmacy in older people—an international consensus study

**DOI:** 10.1093/ageing/afaf102

**Published:** 2025-04-23

**Authors:** Hanadi Al Shaker, Heather Barry, Carmel Hughes

**Affiliations:** School of Pharmacy, Queen’s University Belfast, Belfast, United Kingdom; Faculty of Pharmacy and Medical Sciences, University of Petra, Amman, Jordan; School of Pharmacy, Medical Biology Centre, Queen’s University Belfast , Belfast, United Kingdom; School of Pharmacy, Queen’s University Belfast, Belfast, United Kingdom

**Keywords:** core outcome set, adherence, polypharmacy, older people, outcomes

## Abstract

**Background:**

Medication non-adherence is prevalent in older people taking polypharmacy. Several interventions have been employed to improve adherence in this population. However, inconsistencies in outcomes have impeded comparisons of findings. Accordingly, this work aimed to develop a core outcome set (COS) for use in trials aiming to improve adherence to appropriate polypharmacy in older people.

**Methods:**

A group of stakeholders, including academics, journal editors, healthcare professionals (HCPs) and public participants, evaluated 13 outcomes compiled from the literature in a Delphi study using a nine-point Likert scale ranging from 1 to 9, where higher scores (7–9) indicated critical importance and lower scores (1–3) unimportance. The resultant Delphi consensus list was discussed and voted on (yes: critical and no: unimportant) in two online nominal group technique (NGT) meetings. The NGT followed a five-stage approach: introduction, silent generation, round-robin, clarification and voting. An outcome was included if ≥80% of participants scored it critical and ≤ 15% scored it as unimportant.

**Results:**

Of the 13 outcomes originally presented to participants, consensus was achieved to include six outcomes in the COS after the Delphi study (Round 1, *n* = 57; Round 2, *n* = 53; Round 3, *n* = 50, where ‘n’ represents participant numbers) and the NGT meetings (*n* = 10) comprising medication adherence across multiple medications, treatment burden, health-related quality of life (HRQoL), healthcare utilisation (HCU), adverse events and side effects (AEs and SEs) and cost-effectiveness.

**Conclusion:**

This COS should be used in intervention studies focusing on improving adherence to appropriate polypharmacy in older people. Future work should identify outcome measurement instruments to be used alongside the COS.

## Key Points

- Core outcome sets (COSs) are an agreed list of outcomes that should be reported in all clinical trials on a specific topic.
- Stakeholders advocated the use of six outcomes associated with adherence to appropriate polypharmacy in older people.
- The study followed a stepwise approach in developing a COS focusing on adherence to appropriate polypharmacy in older people.
- Implementing this COS will enhance the comparability of trials focusing on adherence to appropriate polypharmacy in older people.

## Introduction

Globally, the number of older people aged ≥65 years is expected to double from 761 million in 2021 to more than 1.5 billion in 2050 [1]. This change is accompanied by increasing multimorbidity, resulting in around half of the older population receiving polypharmacy [2, 3]. There is no unanimously accepted definition of polypharmacy [4]. However, for the purpose of this study and in line with previous Cochrane reviews, polypharmacy is defined as the concurrent administration of four or more medications [4, 5]. Appropriate polypharmacy refers to prescribing multiple medications for multimorbid patients, where all medications are optimised and prescribed according to the most recent evidence [6, 7]. Efforts to tackle medication adherence to polypharmacy in older people have focused on different behavioural and/or educational interventions [5]. However, a Cochrane review on adherence and medication-taking ability in older adults prescribed polypharmacy revealed significant variability in interventions implemented, outcomes and outcome measures used, resulting in low-quality evidence [5].

The Core Outcome Measures in Effectiveness Trials (COMET) initiative was launched to raise awareness of outcome heterogeneity and encourage relevant stakeholders to develop evidence-based core outcome sets (COSs) [8]. A COS is an agreed list of outcomes of critical importance that should be reported and evaluated in all clinical trials on a specific health topic [8].

No COS has been developed for interventions to enhance older adults’ adherence to appropriate polypharmacy. Therefore, this work aimed to develop such a COS to be used in future clinical trials focusing on this area.

## Methods

Development of the COS followed the COMET initiative recommended approach, registered on the COMET database (https://www.comet-initiative.org/Studies/Details/2698) and reported following the COS—Standards for Reporting guidelines (Appendix 1) [8, 9]. This study involved three phases: Phase 1: compiling and refining a relevant list of outcomes; Phase 2: a Delphi consensus exercise; and Phase 3: an online nominal group technique (NGT) study. The scope of this COS encompassed intervention studies aiming to improve adherence to appropriate polypharmacy (≥4 regular medications) in older people (aged ≥65 years) living in their own homes (i.e. community-dwelling patients) and who can manage their medications.

### Phase 1: Compiling and refining potentially relevant list of outcomes (October 2021–October 2022)

A list of outcomes was compiled from a Cochrane review targeting older people’s adherence to polypharmacy [5], two other studies and semi-structured interviews with key stakeholders focused on outcomes to improve older patients’ adherence to appropriate polypharmacy [10–12].

### Phase 2: The Delphi consensus exercise (February 2023–April 2023)

In Phase 2, a three-round sequential Delphi study was conducted to condense the outcomes compiled following Phase 1. There is no specific guidance concerning the recommended number of participants in a Delphi panel; previous COS studies ranged from 15 participants to hundreds [13]. We anticipated that 100 or more participants would take part in Round 1 of the Delphi study.

Potential participants received invitation emails and study information sheets explaining the study’s aim and process. We sought to recruit experts and public participants from around the world for the Delphi exercise and the NGT meetings. The targeted expert group comprised: (i) academics with expertise in medication adherence, polypharmacy, medication optimisation and/or older people; (ii) healthcare professionals (HCPs) who provide care to older patients (e.g. doctors, pharmacists and nurses) and (iii) editors of peer-reviewed journals focusing on adherence, polypharmacy, ageing, drug safety and gerontology. Academics and HCPs were identified by searching bibliographic databases and journal editors by searching journal editorial boards and journal information (Appendix 2). Public participants were sought by searching public contact information of employees (i.e. email addresses) working in charities/organisations (based in English-speaking countries) concerned with providing care, advice and support and advocating for the well-being of older people in healthcare. These charities/organisations were also asked about their willingness to assist with recruitment. Snowball sampling was also utilised to support recruitment. Delphi questionnaires were piloted with researchers from the School of Pharmacy, Queen’s University Belfast (QUB) and revised based on feedback.

Ethical approval was obtained from the Faculty of Medicine, Health and Life Sciences (FMHLS) Research Ethics Committee, QUB (Reference number: MHLS 23_09). Individuals who agreed to participate were sent a first-round email containing their identification codes and a web-based questionnaire link via an online platform called SoGolytics®. Participants were given an overview of the study and instructions on questionnaire completion.

The first and second parts of the Delphi questionnaire presented a mandatory consent form and demographic details for completion before proceeding to the third part, which comprised the questionnaire. A list of outcomes, along with definitions, was presented to participants to rate according to the COMET recommended scoring scale devised by the Grading of Recommendations, Assessment, Development and Evaluation (GRADE) [14]. Participants scored between 1 and 9 for each outcome depending on their degree of importance from their perspective (1–3, unimportant; 4–6, important but not critical; 7–9, critical). An ‘unable to score’ option was included. In the final part, participants were invited to propose other outcomes not included in the original list of outcomes; these were checked against the original list to eliminate outcomes out of scope or duplication.

There were two weeks between each round, and non-respondents received a reminder after one week to encourage completion. The response rate and percentage of participants who rated each outcome using the above scale were calculated after each round. In Rounds 2 and 3, participants received individualised feedback reports and group feedback (bar charts) to compare their responses with other participants’ views. Round 2 included all outcomes from Round 1 and any additional suggested outcomes accepted by the research team. Round 3 consisted of outcomes that were not agreed upon in the second round.

### Phase 3: The online nominal group technique study (September 2023–October 2023**)**

Following the Delphi study, two online NGTs were held to refine the outcomes list further. Meetings took place at different times to accommodate international participation. We planned to include both experts and public participants in each NGT meeting [15, 16]. Experts who took part in the Delphi study were also invited to participate in the NGT meetings. The NGT does not rely on statistical power [17]; however, 10 is considered the optimal number for the NGT panels [18]. Therefore, we expected to recruit between 10 and 14 individuals for both sessions [18, 19]. Sampling and recruitment of experts and public members were conducted as in the Delphi exercise. The NGT process was held virtually via Microsoft Teams®, and the SoGolytics® platform was used for data collection, following a pilot phase. Ethical approval was granted by the FMHLS Research Ethics Committee, QUB (Reference number: MHLS 23_111).

### The nominal group technique process

The NGT process was guided by a script (Appendix 3) and consisted of five stages: introduction, silent generation, round-robin, clarification and voting [20, 21]. Following the introduction, participants received identification codes and a web-based link containing the NGT workbooks, whereby the Delphi study outcomes, definitions and a text box were presented to record their silent generation thoughts. In the round-robin stage, a report comprising all anonymised responses was shared on screen, and participants provided their perspectives/experiences about each outcome individually. All participants were then invited to discuss/compare perspectives in the clarification stage. Finally, participants received their identification codes and a web-based link containing the voting questionnaire to anonymously vote on outcomes by selecting ‘yes’ and ‘no’ and providing an optional justification when selecting ‘no’. The process is summarised in Appendix 4.

### Data analysis

Based on the COMET initiative recommendations, the scoring system and consensus criteria were set before the Delphi exercise and the NGT study. Table 1 outlines how data analysis was conducted in both phases.

**Table 1:** Data analysis approach for the Delphi consensus study and the NGT meeting.

**Delphi consensus exercise**	The scoring system: The scoring system used a nine-point Likert scoring system, whereby critical outcomes were rated between 7 and 9, and outcomes of limited importance were rated between 1 and 3 [8, 14]. Consensus definition: Round 1 to Round 2: All outcomes were carried over to Round 2. After Rounds 2 and 3: Outcomes rated between 7 and 9 by 80% or more of participants AND between 1 and 3 by 15% or less were included in the list of outcomes (‘consensus in’). Conversely, outcomes rated between 7 and 9 by 15% or less of the participants AND between 1 and 3 by 80% or more were excluded (‘consensus out’). All ‘no consensus’ outcomes were retained. After Round 3: Outcomes that achieved ‘no consensus’ were excluded after Round 3. Accordingly, only ‘consensus in’ outcomes from Round 3 were carried forward to the NGT meeting.
**NGT study**	The scoring system: The scoring system encompassed ‘yes’ and ‘no’ options, whereby the former indicated outcomes of critical importance, whilst the latter denoted outcomes of limited importance. Consensus definition: Similar to the consensus criteria of the Delphi study, outcomes voted on as ‘yes’ by ≥80% of participants AND ‘no’ by ≤15% of participants were included in the final COS. Outcomes voted on as ‘no’ by ≥80% of the participants AND ‘yes’ by ≤15% of participants were excluded from the final COS. Outcomes that achieved ‘no consensus’ were included at the end of the NGT study.

All COS outcomes were classified according to a taxonomy advocated by the COMET initiative [22], encompassing 5 core areas and 38 outcome domains [22].

## Results

### Phase 1: Compiling and refining a potentially relevant list of outcomes

In Phase 1, a list of 13 outcomes, comprising seven outcomes from a Cochrane review of randomised controlled trials [5] and two relevant studies related to adherence to polypharmacy [10, 11], and six outcomes from semi-structured interviews with key stakeholders [12] were compiled and included in the three-round Delphi study (Appendix 5).

### Phase 2: The Delphi consensus exercise

In total, 250 experts and 18 public members were invited to participate. Of these, 55 experts and one public member provided consent and demographic details, with three additional public members recruited via snowball sampling. Twenty-five organisations were also approached to seek assistance in recruitment; two declined the invitation, and no other responses were received. Table 2 outlines response rates and the demographic information of participants.

**Table 2:** Demographic details of participants in the Delphi consensus study and the NGT meetings.

Participant characteristics	Delphi consensus exercise			NGT meetings
	Round 1	Round 2	Round 3	
**Participant, n**	57	53	50	10
**Response rate, %**	96.6	89.8	84.7	76.9
**Age in years, median (range)**	50 (26–87)	50 (26–87)	49 (26–87)	54 (39–60)
**Gender, n (%)**				
Men	21 (36.8)	20 (37.7)	19 (38.0)	1 (10.0)
Women	36 (63.2)	33 (62.3)	31 (62.0)	9 (90.0)
**Continent of residence, n (%)**				
America	8 (14.0)	8 (15.1)	7 (14.0)	1 (10.0)
Europe	39 (68.4)	37 (69.8)	36 (72.0)	8 (80.0)
Australia	8 (14.0)	7 (13.2)	6 (12.0)	1 (10.0)
Asia	2 (3.5)	1 (1.9)	1 (2.0)	0 (0.0)
**Professional area, n (%)** ^a^				
Doctor	12 (21.1)	11 (20.8)	11 (22.0)	1 (10.0)
Pharmacist	23 (40.4)	22 (41.5)	22 (44.0)	5 (50.0)
Academic	38 (67.0)	35 (66.0)	34 (68.0)	10 (100.0)
Journal editor	6 (10.5)	5 (9.4)	5 (10.0)	2 (20.0)
Nurse	3 (5.3)	3 (5.7)	1 (2.0)	1 (10.0)
Public member	4 (7.0)	3 (5.7)	3 (6.0)	0 (0.0)

a The sum of percentages shown in the table exceeds 100% as some participants selected multiple options.

**Figure 1:**
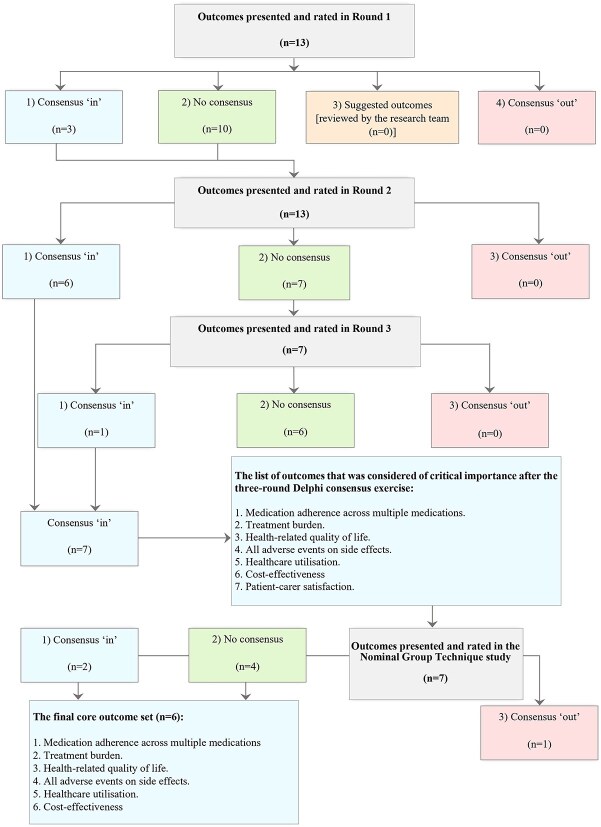
Flow chart outlining the Delphi and the NGT processes.

Of the 59 respondents who agreed to take part, 57 participants (response rate: 96.6%) completed the first questionnaire. Three outcomes achieved consensus for inclusion, with an additional 33 outcomes proposed by participants. Following review, no suggested outcome was deemed relevant to be included in the second Delphi questionnaire. Therefore, all outcomes presented in Round 1 were carried forward to Round 2. In Round 2 (*n* = 53; response rate: 89.8%), six outcomes met the ‘consensus in’ threshold and were included in the list of outcomes. The remaining seven outcomes that achieved ‘no consensus’ were presented in Round 3, in which 50 participants (84.7%) completed the questionnaire. Overall, seven outcomes were considered critical: medication adherence across multiple medications, treatment burden, health-related quality of life (HRQoL), all adverse events and side effects (AEs and SEs), healthcare utilisation (HCU), cost-effectiveness and patient-carer satisfaction (Fig. 1).

The distribution of scores for each outcome in all rounds is outlined in Appendix 6.

### Phase 3: The online nominal group technique study

Initially, 233 potential experts and 18 public members were invited to participate. Nineteen (8.5%) experts accepted the invitation, with no responses from public members. Thirteen respondents completed the demographics and consent forms, but only 10 experts participated (six in one meeting and four in the other), seven of whom had also participated in the Delphi study. These experts were located in America, Australia and Europe; 90% were female (Table 2). Both meetings lasted approximately two hours and 15 minutes.

#### The nominal group technique workbooks

Participants recorded that most outcomes were important and should be included in the final COS, except for patient-carer satisfaction. There were also concerns regarding the inclusion of cost-effectiveness in the list. Table 3 displays all outcomes, along with their explanations and excerpts of the participants’ responses. Silent generation responses are presented in Appendix 7 as recorded in the SoGolytics® platform.

**Table 3:** Participants’ silent generation responses about all outcomes.

**1. Medication adherence across multiple medications**	
**Explanation and response excerpt/s**	All experts considered medication adherence across multiple medications the primary and core outcome of this study. “*YES—This should be included as it is central to clinical trials aimed at identifying interventions to improve adherence to appropriate polypharmacy.*” [P9] (P9 is a pharmacist and an academic) Nevertheless, they believed there was a lack of consensus on the appropriate method of measuring adherence to many medicines. “*There is currently no “gold standard” approach to measuring adherence across multiple medicines.*” [P1] (P1 is a pharmacist, an academic and a journal editor)
**2. Treatment burden**	
**Explanation and response excerpt/s**	Participants unanimously deemed treatment burden a significant outcome influencing patients’ non-adherence to multiple medications, especially when older patients take polypharmacy. “*Yes very important. Polypharmacy poses significant burden on patients. If they are experience [sic] burden—be it physical, mental of financial, then they are likely to stop adhering to meds.*” [P2] (P2 is an academic) Furthermore, participants reported that measuring treatment burden was difficult because the type of intervention used should be specified, and a consistent method should be adopted to ensure accurate measurement. “*Again, if we keep it, standardising assessment of treatment burden would be key in my opinion to have a valuable, comparable indicator.*” [P3] (P3 is a pharmacist and an academic) “*Important to assess as overall treatment burden and the impact of an intervention in this regard.*” [P4] (P4 is an academic) “*It is also very difficult to measure.*” [P5] (P5 is a nurse and an academic)
**3. Health-related quality of life**	
**Explanation and illustrative example**	HRQoL was deemed a significant measure. Nevertheless, this outcome was believed to be less responsive and unlikely to show any intervention-related differences in adherence improvement in long-term follow-up (e.g. six months). “*. . .generic QoL instruments may not be sensitive enough to capture changes in QoL resulting from small increases/decreases in medication adherence.*” [P4] (P4 is an academic) “*Yes and no! I think that quality of life is a really important concept but the measures we have are so insensitive to change in these types of trials that it is meaningless.*” [P2] (P2 is an academic) Furthermore, participants believed that most existing HRQoL measures were subjective and self-reported, imposing a response burden on participants and increasing self-reporting errors. “*It creates burden [sic] on participants to have to fill in these questionnaires when they are somewhat meaningless.*” [P2] (P2 is an academic)
**4. All adverse events and side effects**	
**Explanation and illustrative example**	Participants emphasised that all adverse events and side effects should be included in the COS, given the potential for polypharmacy to cause negative consequences and complications that affect older people’s health and safety. “*There is significant [sic] burden associated with polypharmacy so avoiding the adverse effects is crucial to avoid unnecessary harm caused by medicines.*” [P6] (P6 is an academic) Participants reported that it was challenging to measure all adverse events and side effects due to their wide-ranging nature, complexity and the difficulty in differentiating whether these side effects and/or adverse events were caused by the disease symptoms of a disease (e.g. cancer) or the treatment itself. “*If we want to fully understand drug interactions, prescribing cascades etc in real patients then we should be collecting this data*” [P2] (P2 is an academic) “*. . .it is not always possible to connect one medication to one side-effect.*” [P5] (P5 is a nurse and an academic)
**5. Healthcare utilisation**	
**Explanation and illustrative example**	Although healthcare utilisation was believed to be linked to the cost-effectiveness analysis, this outcome was deemed multidimensional and complex, impacted by several factors (e.g. financial stability, education). “*No, I would not include the outcome in the COS. Healthcare utilisation is influenced by so many factors, various in different countries, depending on health literacy, steady income and many more aspects. It is not suitable for a COS*” [P5] (P5 is a nurse and an academic) “*yes [sic], esp [especially] related to outcome 7 [cost-effectiveness].*” [P8] (P8 is a pharmacist and an academic) Moreover, participants noted that the type of healthcare services measured would impact healthcare utilisation, such as hospital admission, emergency department visits and pharmacy services. “*This depends on what we will measure: pharmacy services, ambulance, paramedic, emergency department visits, hospitalisations*” [P1] (P1 is a pharmacist, an academic and a journal editor)
**6. Cost-effectiveness**	
**Explanation and illustrative example**	Participants expressed conflicting opinions concerning the inclusion of cost-effectiveness in the COS. Some participants thought this outcome should be included to convince decision-makers and policymakers to implement a new intervention. “*Yes. If we want to implement something, we need to know that it is effective, safe and cost-effective. That is what drives policy change so we must have this.*” [P2] (P2 is an academic) “*Yes. Important to support decision making in terms commissioning and role [sic] out of successful interventions in routine practice*” [P4] (P4 is an academic) In contrast, others believed that measures used to evaluate and report cost-effectiveness in previous international studies were inconsistent. Participants also noted the relationship between cost-effectiveness with HRQoL and healthcare utilisation. "No. The measurability in different countries is questionable and there are probably very different aspects, and it needs to be discussed what is realy [sic] cost-effectiveness in consideration for example to qualtiy [sic] of live" [P5] (P5 is a nurse and an academic) “*No. Although I think this would be important to include and relates to some of the other outcome measures already mentioned e.g. healthcare utilisation, HRQoL*” [P6] (P6 is an academic)
**7. Patient-carer satisfaction**	
**Explanation and illustrative example**	Participants acknowledged that patients’ and carers’ satisfaction with healthcare services reflected patients’ satisfaction with the overall quality provided. However, they believed that patient and/or carers’ satisfaction should be collected as information for intervention process evaluation (e.g. acceptability with the intervention) rather than an outcome per se. “*I am mixed on this one, but no. I think overall, very important to understand patient/carer experiences of health care but perhaps more qualitatively, something to explore in a process evaluation.*” [P2] (P2 is an academic) “*no [sic] or mixed, but mostly in terms of acceptability of an adherence intervention.*” [P8] (P8 is a pharmacist and an academic) In addition, participants stated that measuring satisfaction mostly depended on subjective self-reported questionnaires, imposing an additional burden on patients. “*Also, it will be a self-reported subjective measure which is less reliable as an outcome in a trial.*” [P6] (P6 is an academic)

COS, core outcome set; HRQoL: health-related quality of Life; QOL, quality of life.

#### The nominal group technique voting questionnaires

All 10 participants completed the voting questionnaires after the round-robin and group discussion stages ended. Two outcomes achieved the ‘consensus in’ threshold and were included in the final COS: medication adherence across multiple medications and AEs and SEs. Conversely, four outcomes achieved the ‘no consensus’ threshold: treatment burden, cost-effectiveness, HCU and HRQoL. One outcome, patient-carer satisfaction, was excluded from the final COS (Figure 1).

Consistent with our consensus criteria, all ‘no consensus’ outcomes were included in the final COS, resulting in a total of six outcomes, which were classified into core areas and domains according to the Dodd et al. taxonomy [22]. Table 4 displays the core areas, outcome domains and outcomes, along with the degree of importance for each outcome.

**Table 4:** Degree of importance for each outcome based on the scale used in the NGT study.

Core area	Outcome domain	Outcomes	Rating by 10 participants, n (%)		Consensus results according to the consensus criteria used (80%/15%)^a^
			Yes (important and should be included in the final COS)	No (unimportant and should not be included in the final COS)	
Life impact	Global quality of life	HRQoL	6 (60.0)	4 (40.0)	No consensus
	Delivery of care	Medication adherence across multiple medications	10 (100.0)	0 (0.0)	Consensus in
		Treatment burden	8 (80.0)	2 (20.0)	No consensus
Resource use	Resource use	Cost-effectiveness	4 (40.0)	6 (60.0)	No consensus
		Healthcare utilisation	8 (80.0)	2 (20.0)	No consensus
Adverse events	Adverse events/effects	All adverse events and side effects	9 (90.0)	1 (10.0)	Consensus in
-	-	Patient-carer satisfaction	1 (10.0)	9 (90.0)	Consensus out (excluded)

a Outcomes voted on as ‘yes’ by ≥80% of the participants AND ‘no’ by ≤15% of the participants were included in the final COS, whereas outcomes voted on as ‘no’ by ≥80% of the participants AND ‘yes’ by ≤15% of the participants were excluded from the final COS. Outcomes which achieved ‘no consensus’ were included at the end of the NGT study. ‘Consensus out’ outcomes were excluded from the final list of outcomes.

## Discussion

This international consensus-based work has led to the development of a COS for use in trials aiming to improve adherence to appropriate polypharmacy in older people. Seven outcomes were deemed important for inclusion after the Delphi study and were discussed in the NGT meetings, including medication adherence across multiple medications, AEs and SEs, HRQoL, treatment burden, HCU, cost-effectiveness and patient-carer satisfaction. The remaining six outcomes that were neither ‘consensus in’ nor ‘consensus out’ following the Delphi study were excluded: mortality, condition-specific outcomes, medication wastage, frailty, falls and HRQoL for caregivers. Patient-carer satisfaction was subsequently omitted after the NGT meetings (i.e. consensus out). Accordingly, the final COS comprised six outcomes from three core areas: resource use, adverse events and life impact, with the latter two core areas proven to be less frequently reported in earlier COS studies [22].

Unsurprisingly, medication adherence across multiple medications and treatment burden received unanimous support in the Delphi questionnaires and NGT meetings. Despite being a core outcome in this COS, many participants believed measuring medication adherence to multiple medications was challenging. Indeed, a systematic review concluded that the high number of prescribed medications, study design (e.g. prospective and retrospective study), the type of chronic conditions (e.g. cardiovascular disease, mental health conditions) and variability of measurement instruments (e.g. self-reported questionnaires) used to measure adherence to polypharmacy reinforced this difficulty [23].

Similarly, participants highlighted that assessing treatment burden was challenging due to the difficulty in applying standard and reliable measures, as well as the multidimensional components affecting this outcome. This is underscored by the absence of universally accepted guidance on methods employed to measure treatment burden [24].

Although participants supported the inclusion of HRQoL in the Delphi study and the consensus meetings, concerns were raised in the NGT meetings about its subjective nature and insensitivity to change. Cross *et al.* [5] confirmed that no previous interventions related to improving adherence to polypharmacy in older patients had significantly affected HRQoL compared to usual care. This could be ascribed to differences in the quality of measurements (e.g. validity, reliability) and types of questionnaires used (e.g. generic or disease-specific) to assess HRQoL [5, 25].

Participating experts acknowledged the importance of AEs and SEs in older patients receiving polypharmacy, but noted that measuring these outcomes was challenging due to factors that needed to be considered alongside polypharmacy (e.g. medication-related harm). They also emphasised the difficulty of discerning whether a treatment/intervention or a chronic condition led to AEs and SEs. The latter could arise due to age-related changes (i.e. cognitive, physiological, pharmacokinetics) and the fragmented multi-agency care across the health and social care systems [26, 27].

Furthermore, some participants highlighted that HCU could be influenced by factors such as health literacy and financial stability. Indeed, Andersen’s behavioural model explains the multidimensional factors affecting patients’ decision-making regarding access and utilisation of healthcare services. This model posits that patients’ decision-making is an interaction between enabling (e.g. financial status), predisposing (e.g. age, education) and need factors (e.g. disease symptoms) [28–30].

Although some experts wished to include cost-effectiveness, others reported discrepancies in methods employed to evaluate it due to several non-health (e.g. patient transportation) and health-related factors (e.g. disease severity) [31, 32].

Participants also indicated that patient-carer satisfaction was employed to identify areas of improvement and overall healthcare service satisfaction. However, they highlighted that satisfaction was measured as part of a process evaluation to assess intervention implementation and acceptance rather than being a key outcome. Patient-carer satisfaction was subsequently excluded following the voting stage.

Although this work sought to develop a COS for clinical trials aimed at improving adherence to appropriate polypharmacy in older people, it can also be used for routine monitoring in primary care. In addition, it is hoped that this COS will be incorporated into future guidelines concerning improving adherence to multiple medications and chronic conditions management, such as those produced by the World Health Organization. Journals concerned with ageing-related interventions should also encourage the adoption of the COS in future published clinical trials to harmonise research data and facilitate intervention implementation. Finally, in order to promote the uptake of this COS and reduce research waste, research funders could integrate explicit requirements that mandate trialists to search for an existing COS related to their area of interest (i.e. adherence to appropriate polypharmacy in older patients). Otherwise, trialists should state the rationale for selecting different outcome sets. Funders could also cross-check the existence of a COS in the COMET database before funding approval, thus, enhancing transparency of outcome selection in clinical practise [33].

This study had several strengths. The Delphi panel consisted of diverse international stakeholder groups, including HCPs with different backgrounds (e.g. pharmacists, doctors and nurses), journal editors and academics with expertise in research targeting polypharmacy, older people and adherence. Similarly, NGT participants were dispersed over seven countries, which may increase the generalisability of results. Another strength was the high response rates across the three Delphi rounds (96.6%, 89.8% and 84.7%, respectively). Third, due to the selection of stringent consensus criteria (‘80%/15%’), only outcomes believed to be highly important were included in the Delphi study. However, in the NGT study, this consensus criterion resulted in a higher number of outcomes that met the ‘no consensus’ threshold (n = 4), owing to the small number of participants who took part (n = 10). We suggest that future research reduces the difference between the ‘consensus in’ and ‘consensus out’ thresholds to minimise the occurrence of ‘no consensus’ outcomes (e.g. ‘80%/20%’). In addition, the NGT meetings ensured that all experts’ perspectives (HCPs, academics and journal editors) were considered equally. The NGT process also allowed experts to think about each outcome, document and discuss their thoughts with other colleagues. Finally, the number of experts involved in both NGT meetings was in line with the number of participants recommended for such studies [18].

A major limitation of this study was the small number of included public participants despite approaching relevant older people’s organisations and charities and implementing the snowball sampling technique. Although we aimed to recruit experts from all countries worldwide, we only approached older people’s organisations and charities in English-speaking countries to avoid translation for which there was no support. Consequently, there were no public participants and few experts from Africa, Asia and South America. Future COS studies should identify pragmatic solutions to facilitate international patient and public involvement in COS development research by using translated questionnaires and employing multilingual NGT moderators/facilitators. Furthermore, the number of included participants in the Delphi study was lower than the anticipated targeted sample size (n = 100).

A list of six outcomes has been developed for trials evaluating interventions focusing on adherence to appropriate polypharmacy in older people. This COS could standardise outcome selection and reporting and improve the quality of evidence in meta-analyses and systematic reviews. Future work will select outcome measurement instruments for outcomes included in this COS to ensure their feasible applicability across various healthcare settings.

## Supplementary Material

aa-24-2772-File003_afaf102

## Data Availability

Pseudonymised datasets generated during and/or analysed during the current study are available from the corresponding author on reasonable request.
