# Supplementary material for: Development of a core outcome set for clinical trials targeting interventions aiming to improve adherence to appropriate polypharmacy in older people—an international consensus study
Source: Age Ageing. 2025 Apr 23;54(4):afaf102. doi: 10.1093/ageing/afaf102 (PMC12017393; doi:10.1093/ageing/afaf102)
Supplement: aa-24-2772-File003_afaf102 [file aa-24-2772-file003_afaf102.pdf]

Online Supplementary Material

Development of a core outcome set for clinical trials targeting interventions aiming to improve adherence to appropriate polypharmacy in older people - an international consensus study

Table of Contents

Appendix 1. Core Outcome Set-STandards for Reporting: The COS-STAR Statement Nominal group technique: a users' guide 1

Appendix 2. A list of journals from which editors were identified and older people's organisations and charities contacted to promote public participation 3

Appendix 3. NGT script for academics, healthcare professionals, journal editors and public members 6

Appendix 4. The online NGT meeting overview 12

Appendix 5. The list of outcomes and corresponding definitions presented to participants in the Delphi questionnaires 13

Appendix 6. Degree of importance for each outcome following Rounds 1, 2 and 3 of the Delphi consensus exercise 16

Appendix 7. Participants' silent generation responses about the outcomes resulting from the nominal group consensus meetings 18

Appendix 1. Core Outcome Set-STandards for Reporting: The COS-STAR Statement Nominal group technique: a users' guide

SECTION/TOPIC	ITEM No.	CHECKLIST ITEM	REPORTED ON PAGE NUMBER
**TITLE/ABSTRACT**			
Title	1a	Identify in the title that the paper reports the development of a COS	1
Abstract	1b	Provide a structured summary	1–Abstract
**INTRODUCTION**			
Background and Objectives	2a	Describe the background and explain the rationale for developing the COS.	2
	2b	Describe the specific objectives with reference to developing a COS.	1–2
Scope	3a	Describe the health condition(s) and population(s) covered by the COS.	3
	3b	Describe the intervention(s) covered by the COS.	3
	3c	Describe the setting(s) in which the COS is to be applied.	3
**METHODS**			
Protocol/Registry Entry	4	Indicate where the COS development protocol can be accessed, if available, and/or the study registration details.	2
Participants	5	Describe the rationale for stakeholder groups involved in the COS development process, eligibility criteria for participants from each group, and a description of how the individuals involved were identified.	3–4
Information Sources	6a	Describe the information sources used to identify an initial list of outcomes.	3
	6b	Describe how outcomes were dropped/combined, with reasons (if applicable).	6–8, Appendix 6 and Table 4
Consensus Process	7	Describe how the consensus process was undertaken.	4–8
Outcome Scoring	8	Describe how outcomes were scored and how scores were summarised.	4–8<br>Table 1
Consensus Definition	9a	Describe the consensus definition.	Table 1
	9b	Describe the procedure for determining how outcomes were included or excluded from	4–7<br>Table 1

SECTION/TOPIC	ITEM No.	CHECKLIST ITEM	REPORTED ON PAGE NUMBER
		consideration during the consensus process.	
Ethics and Consent	10	Provide a statement regarding the ethics and consent issues for the study.	4 and 5
**RESULTS**			
Protocol Deviations	11	Describe any changes from the protocol (if applicable), with reasons, and describe what impact these changes have on the results.	Not applicable
Participants	12	Present data on the number and relevant characteristics of the people involved at all stages of COS development.	Table 2
Outcomes	13a	List all outcomes considered at the start of the consensus process.	Appendix 5
	13b	Describe any new outcomes introduced and any outcomes dropped, with reasons, during the consensus process.	6–8, Appendix 6
COS	14	List the outcomes in the final COS.	Table 4
**DISCUSSION**			
Limitations	15	Discuss any limitations in the COS development process.	11
Conclusions	16	Provide an interpretation of the final COS in the context of other evidence, and implications for future research.	10 and 11
**OTHER INFORMATION**			
Funding	17	Describe sources of funding/role of funders.	Title page
Conflicts of Interest	18	Describe any conflicts of interest within the study team and how these were managed.	Title page

From: Kirkham JJ, Gorst S, Altman DG, Blazeby JM, Clarke M, Devane D, et al. (2016) Core Outcome Set–STAndards for Reporting: The COS-STAR Statement. PLoS Med 13(10): e1002148. https://doi.org/10.1371/journal.pmed.1002148

Appendix 2. A list of journals from which editors were identified and older people's organisations and charities contacted to promote public participation

Journal editors:

A list of journals concerned with adherence, patient education, drug safety, polypharmacy, ageing and gerontology from which editors were identified:

1) Age and Ageing	17) Drug Safety	33) Journal of Aging Studies
2) Ageing & Society	18) Drugs & Aging	34) Journal of Anti-Aging Medicine
3) Ageing International	19) European Geriatric Medicine	35) Journal of Family Medicine and Primary Care
4) Ageing Research Reviews	20) European Journal of Ageing	36) Journal of Integrated Care
5) Aging and Disease	21) Experimental Aging Research	37) Journal of the American Geriatrics Society
6) Aging and Mental Health	22) Experimental Gerontology	38) Journal of Women & Aging
7) Aging Clinical and Experimental Research	23) Geriatric Nursing	39) Patient Education and Counselling
8) Aging Medicine and Healthcare	24) Geriatrics	40) Patient Preference and Adherence
9) Aging, Neuropsychology, and Cognition	25) GeroScience	41) Quality in Ageing and Older Adults
10) Aging-US	26) International Journal of Alzheimer's Disease	42) Research on Aging
11) BMC Geriatrics	27) International Journal of Integrated Care	43) The International Journal of Aging and Human Development
12) Canadian Geriatrics Journal	28) International Journal of Older People Nursing	44) The Journal of Nutrition, Health & Aging
13) Clinical Interventions in Aging	29) Journal of Aging & Social Policy	45) The Lancet Healthy Longevity
14) Clinics in Geriatric Medicine	30) Journal of Aging and Health	46) Therapeutic Advances in Drug Safety
15) Current Aging Science	31) Journal of Aging and Physical Activity	
16) Current Geriatrics Reports	32) Journal of Aging Research	

Public participants:

Older people's organisations and charities contacted to promote public participation in the study:

The United Kingdom (UK):

1. Involve https://www.involve.org.uk/
2. The National Care Association https://nationalcareassociation.org.uk/
3. British Geriatrics Society https://www.bgs.org.uk/
4. Re-engage https://www.reengage.org.uk/
5. Engage with Age https://engagewithage.org.uk/
6. The University of the Third Age https://www.u3a.org.uk/
7. Volunteer Now NI https://www.volunteernow.co.uk/
8. AgeUK https://www.ageuk.org.uk/
9. Scottish Older People's Assembly http://www.scotopa.org.uk/aboutus.asp

Australia:

1. Consumers Health Forum https://chf.org.au/
2. National Seniors Australia https://nationalseniors.com.au/

The United States:

1. International Association for Public Participation https://www.iap2usa.org/
2. National Alliance for Caregiving https://www.caregiving.org/about/

Canada:

1. Carers Canada https://www.carerscanada.ca/
2. Age-well https://agewell-nce.ca/
3. The Canadian Association on Gerontology https://cagacg.ca/
4. Canadian Longitudinal Study on Aging (CLSA) https://www.clsa-elcv.ca/

Ireland:

1. The Irish Platform for Patient Organisations, Science and Industry https://www.ipposi.ie/
2. Friends of the Elderly https://friendsoftheelderly.ie/
3. Age Action Ireland https://www.ageaction.ie/
4. Family Carers Ireland https://familycarers.ie/
5. Care Alliance Ireland https://www.carealliance.ie/index

Europe:

1. Eurocarers https://eurocarers.org/
2. European Institute for Public Participation https://participedia.net/organization/201#

Appendix 3. NGT script for academics, healthcare professionals, journal editors and public members

1. <u>Pre-elicitation or introduction stage</u>

Good morning/afternoon. My name is Hanadi, and I'm a PhD research student from the School of Pharmacy, Queen's University Belfast. I'll be moderating today's session. Thank you for taking the time to participate in this meeting. I greatly appreciate it.

As you are aware, this study is a part of a project which hopes to identify a list of important outcomes that can be measured in future trials evaluating interventions focusing on adherence to appropriate polypharmacy. This list of important outcomes is called a Core Outcome Set (COS).

We've done two studies so far. In our first study, a group of outcomes was compiled from other research, which were then discussed with key stakeholders during a semi-structured interview. The key stakeholders agreed that 13 outcomes seemed to be important. In the second study, these 13 outcomes were included in the Delphi consensus exercise you had participated in. The Delphi consensus study aimed to reach agreement on the most important outcomes for a COS. Academics, healthcare professionals, journal editors and public members completed a series of three online questionnaires, which included the outcomes. As a result, seven outcomes from the 13 presented outcomes reached consensus from the Delphi study, namely 1) medication adherence across multiple medications, 2) treatment burden, 3) health-related quality of life, 4) all adverse events or side effects, 5) healthcare utilisation, 6) cost-effectiveness and 7) patient-carer satisfaction.

This third study, which will use the Nominal Group Technique, aims to refine and finalise the list of outcomes obtained from the Delphi study to be measured in all future trials assessing if an intervention has improved adherence to appropriate polypharmacy.

Our Nominal Group consensus meeting should last approximately two hours and 30 minutes. Before we kick off, I'd like to emphasise the importance of open conversation. Please feel free to express your opinions and thoughts, ask any questions or provide comments; this will

greatly contribute to our discussion. Please be aware that despite interacting with each other during the meeting, all participants' identities won't be disclosed when outcomes are rated and the final consensus is reached. Everything discussed here is confidential; therefore, no participants' identities should be revealed outside this meeting. In our discussion today, it's important to note that there are no wrong or right responses, all perspectives are valued, and all points of view are important to us.

To give you a general idea about today's meeting, I'll briefly describe how this meeting will run. It will consist of four stages. The first stage is the silent generation of ideas, whereby I'll send an email, containing a link to a workbook embedded in an online system called Sogolytics®. In this workbook, all outcomes will be presented in turn, along with their definition and a text box to record your views on each outcome. You'll be asked about the importance of each outcome and to provide a reason for your answer. You'll be given 25-30 minutes to reflect upon all outcomes silently and write down these reflections in the workbook. When you've finished making your notes, you'll be asked to submit your notes in the workbook on the Sogolytics® system.

After this silent generation stage, I'll prepare a report of all the anonymised responses and ideas recorded in the workbooks, whereby no participants' ID will be revealed during the next stage, which is the round-robin stage. I'll share this report on-screen, and each participant, in turn, will be asked to express and share their views about each outcome, one at a time. Please be aware that no discussion will occur at this stage to give everyone an opportunity to express their views about each outcome without interruptions. This stage is expected to take 5-10 minutes.

Is that okay with you? Are there any questions before moving on to the next stage?

[*Wait for participants' responses*].

In the next stage, which will take the form of a group discussion, all participants will discuss the ideas expressed in the round-robin stage, in which you can compare your views about

each outcome by considering the views expressed by other participants. This stage will take approximately 10-15 minutes.

In the final voting or ranking stage, I'll send each participant a link to a questionnaire platform containing the list of the seven outcomes, which you'll be asked to rank to indicate if you think each outcome should be included in the COS. You need to click on the link and rank each of the seven outcomes discussed earlier by choosing 'yes' or 'no'. It would be very helpful if you could please provide a brief explanation if you select 'no' for any outcome. Is that clear to you? Would you like me to repeat any information?

[*Wait for participants' responses*].

2. <u>Silent generation of ideas</u>

To start off, I'll now send you an email containing a link to the workbook, and your identification code. Please complete the workbook by considering each outcome in turn. Please take your time to think and type your responses about each outcome. If you require any assistance or have any questions, I'd be happy to help. Please submit your responses in the workbook when you're finished. I'd also be grateful if you could all mute your mics at this time.

[*Send the NGT workbook to participants*].

[*Give participants time to complete their responses and answer any questions*].

Thank you very much for taking the time to complete the workbook and submitting your responses. Give me a few minutes to prepare the report, which I'll share on the screen.

[*Prepare the report and share it on-screen*].

Thank you all for providing your responses. Now, let's move on to the next stage.

3. <u>Round-robin or idea-sharing</u>

Your silent reflection notes are now shared on-screen. I'd like to ask each of you, in turn, to explain your views on each outcome. We'll continue until all of you have the opportunity to talk about the outcomes in the list. Please listen to other participants' perspectives about each outcome. You might find it helpful to make notes during this stage, as these could be used in the group discussion, which will follow in the upcoming group discussion stage.

[*Participant 1*], let's start with your responses. Could you please let us know what you think about each of the outcomes presented and whether they are important or not? Which outcomes do you think should be included or taken out? Let's start with [*Outcome 1*].

[*Give Participant 1 time to respond and share his/her views about all outcomes*].

[*Continue until all participants have shared their thoughts*].

Thank you all for your valuable input and for sharing your views about the importance of each outcome in the list. You'll be given a 10-minute break before the next group discussion stage.

4. <u>Clarification or group discussion</u>

Welcome back! Thank you once again for your valuable input and for sharing your views about the importance of each outcome in the list. Now, let's move on to the group discussion stage. So, I'm inviting you all to reflect on the perspectives shared by your fellow participants. Would any of you like to comment on what's been previously raised in the previous stage?

[*Wait for participants' responses*].

Follow-up questions used <u>if</u> needed

- Would you like to respond to one point or more raised by any of the participants?
- Would you like to clarify your own responses further?
- Does anyone have any additional comments?

[*Wait for participants' responses*].

5. <u>Voting</u>

Thank you, everyone, for your comments on all outcomes. Now let's move on to the last stage, the voting stage. I'll send on an email to each of you with your identification code and a link to the questionnaire platform containing the list of all outcomes to rank. Please click on the link and decide if each of the seven outcomes should be included in the COS by indicating 'yes' or 'no'. I'd be grateful if you could briefly explain why you select 'no'. A text box will only pop up when you select 'no', not if you select 'yes'. Your voting will determine and help us finalise the list of outcomes that should be used in intervention studies aiming to improve adherence to appropriate polypharmacy.

Please note that if you select 'Yes', this means the outcome is of critical importance and should be included in the COS, whereas if you select 'No', this means that the outcome is of limited importance and shouldn't be included in the COS. For an outcome to be included in the COS, a consensus (agreement) will be achieved when it's been given a rating of 'Yes' by 80% or more of participants who've completed the questionnaire and 'No' by less than 15% of those who've completed it. Similarly, if an outcome is rated 'No' by 80% or more of the participants and 'Yes' by 15% or less of the participants, it will be excluded from the final COS. Should you have any questions, please don't hesitate to ask.

[*Send the voting questionnaire to participants*].

[*Wait for participants to rate all outcomes*].

6. <u>Closing the NGT consensus meeting</u>

This brings us to the end of today's meeting. Does anyone have any additional comments that you'd like to make? Or any final questions?

[*Wait for participants' responses and answer any questions*].

Consensus meeting 1

Before we wrap up, I’d like to let you know that another meeting such as this will be held. Once the second meeting is concluded, I’ll send you a link containing a report with the final list of outcomes that have been agreed upon by all participants in the two meetings.

Once again, many thanks for your participation in this study.

Consensus meeting 2

Before we wrap up, I’d like to let you know that based on the results from this meeting and an earlier meeting which took place on [Insert day], I’ll send you a link containing a report with the final list of outcomes that have been agreed by all participants in the two meetings.

Once again, many thanks for your participation in this study.

Appendix 4. The online NGT meeting overview

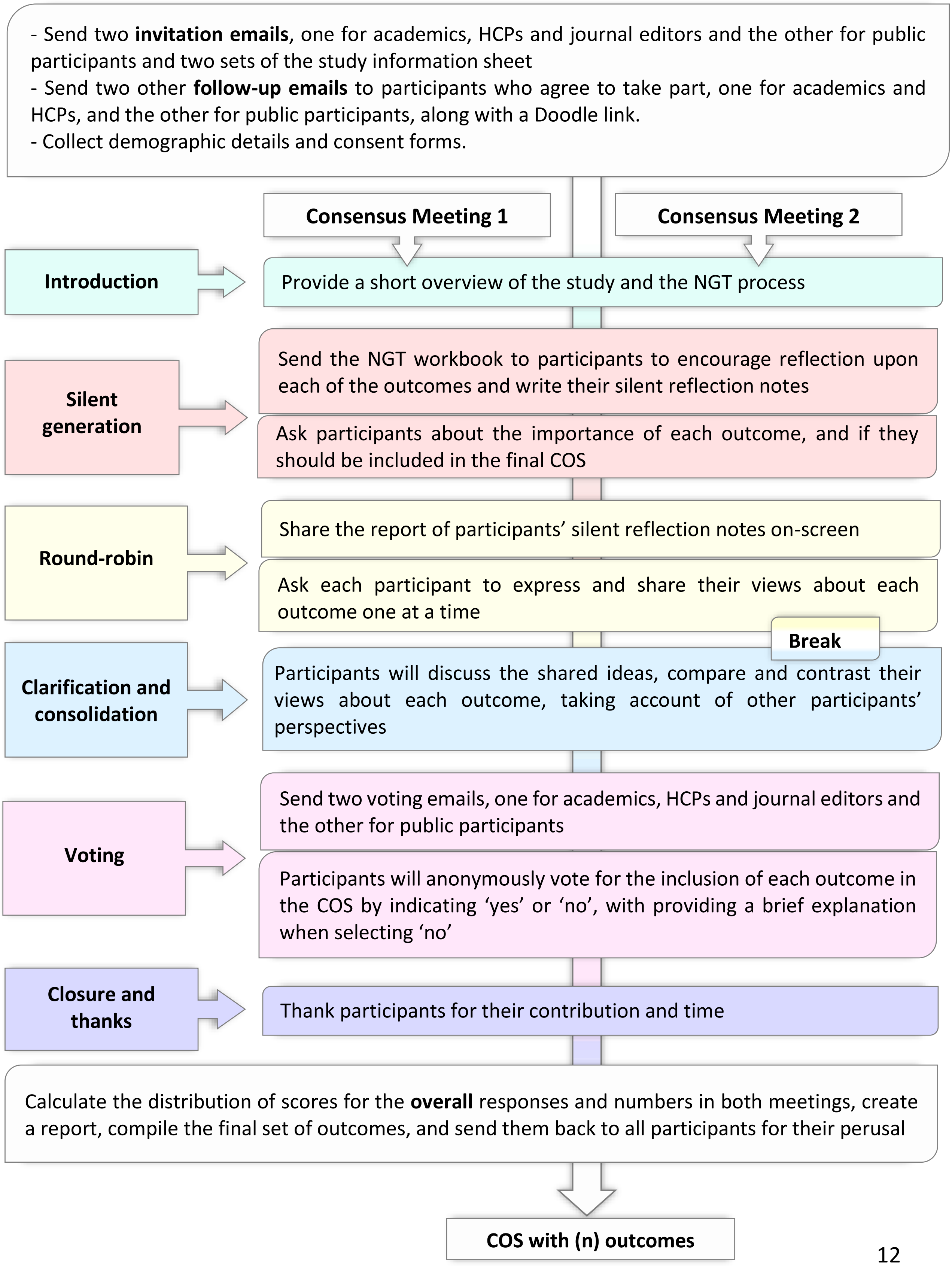

Appendix 5. The list of outcomes and corresponding definitions presented to participants in the Delphi questionnaires

Outcomes	Definitions for academics, HCPs and journal editors	Definitions for public participants
**Medication adherence across multiple medications**	Refers to the degree to which patients take their medicines as advised by HCPs.	Refers to whether patients take all their prescribed medications as directed by their doctor or pharmacist.
**Condition-specific outcomes**	Outcomes related to physiological function, laboratory measurements or clinical assessments for a specific medical condition.	A measurement of some bodily function, such as blood pressure which is measured to see if a medication has lowered blood pressure.
**Mortality**	Death attributed to any reason.	Death due to any reason.
**Treatment burden**	The impact of healthcare on patients' functioning and well-being, apart from specific treatment side effects. It includes visits to HCPs, medical tests, treatment management, and lifestyle changes.	This relates to the activities that people with long-term medical conditions must carry out as directed by HCPs and which can affect their well-being. It includes mental and physical efforts, out-of-pocket costs, and time needed to carry out medical care and healthcare tasks, such as self-monitoring, laboratory tests, doctor visits, and managing medication.
**Falls**	A sudden falling to rest on the ground or lower level due to an unexpected event.	Unexpectedly falling to the ground or lower level.
**Frailty**	A biologic syndrome characterised by older people's vulnerability and loss of physiologic reserve. It has been linked to poor health outcomes, such as	Frailty is the loss of older people's in-built reserve (physical and mental resilience), leading to the possibility of increased falling, moving into a nursing home, loss of

Outcomes	Definitions for academics, HCPs and journal editors	Definitions for public participants
	disability, falls, and hospitalisation.	independence, going into hospital, and dying prematurely.
**All adverse events and side effects**	An unwanted effect caused by a treatment, procedure, or a medicine (e.g. adverse reactions, harm, toxicity, complications).	An unwanted effect or a problem that happens because of treatment, a procedure or a medication.
**Medication wastage**	Any unwanted medication product that is unused, expired, or not fully consumed.	Any unwanted medication product that is unused, out of date, or not fully consumed.
**Health-related quality of life**	A measure of a person's function in life. It evaluates the perceived well-being in mental, physical and social aspects of health.	The extent to which a person is comfortable, healthy, and enjoys life's events.
**Health-related quality of life for caregivers**	A measure of health-related quality of life for people taking care of or supporting a family member, a friend, or a neighbour who has health problems. It evaluates mental, physical, emotional, psychological, social functioning and financial problems.	The effect of caring for an older patient on the lives and health of caregivers and/or family members (e.g. psychological health, time, cost of care).
**Patient/carer satisfaction**	The degree to which a patient and/or carer is satisfied with healthcare quality.	Measures if patients and/or carer are happy with the quality of healthcare services they receive (e.g. quality of care received from a doctor or during a stay in hospital).
**Healthcare utilisation**	The measurement or description of service utilisation by individuals to cure and prevent health conditions, obtaining health information and prognosis, or maintaining patients'	Measures how often a patient uses some kind of service related to their health (e.g. the number of times a patient visits their GP).

Outcomes	Definitions for academics, HCPs and journal editors	Definitions for public participants
	health and well-being (e.g. general practitioners' visits, hospital outpatient clinic visits, community pharmacy visits, inpatient use, emergency department visits, and hospitalisation).	
**Cost-effectiveness**	An indicator to measure costs and health outcomes related to an intervention, e.g. comparing a new service to usual care and estimating how much it costs to gain a unit of health outcome, such as reduced hospital admissions.	Compares the cost of an intervention (e.g. treatment, action, or method carried out to treat, prevent medical conditions and help people stay healthy or improve their health) to see how it affects a patient's health in order to decide if the intervention can be considered value for money.

Appendix 6. Degree of importance for each outcome following Rounds 1, 2 and 3 of the Delphi consensus exercise

Round 1				
**Outcome (n=13)**	**Rating by 57 participants, n (%)**			
	**Critical**	**Important but not critical**	**Not important**	**Unable to score**
**Medication adherence across multiple medications**	**56 (98.2)***	1 (1.8)	0 (0.0)	0 (0.0)
Condition-specific outcomes	38 (66.7)	17 (29.8)	2 (3.5)	0 (0.0)
Mortality	29 (50.9)	22 (38.6)	5 (8.7)	1 (1.8)
**Treatment burden**	**51 (89.5)***	6 (10.5)	0 (0.0)	0 (0.0)
Falls	31 (54.4)	24 (42.1)	2 (3.5)	0 (0.0)
Frailty	30 (52.6)	21 (36.8)	5 (8.8)	1 (1.8)
All adverse events and side effects	44 (77.2)	12 (21.0)	0 (0.0)	1 (1.8)
Medication wastage	22 (38.6)	27 (47.3)	7 (12.3)	1 (1.8)
**Health-related quality of life**	**50 (87.7)***	7 (12.3)	0 (0.0)	0 (0.0)
Health-related quality of life for caregivers	29 (50.9)	24 (42.1)	4 (7.0%)	0 (0.0)
Patient-carer satisfaction	37 (64.9)	16 (28.1)	4 (7.0%)	0 (0.0)
Healthcare utilisation	41 (71.9)	16 (28.1)	0 (0.0)	0 (0.0)
Cost-effectiveness	40 (70.1)	14 (24.6)	2 (3.5)	1 (1.8)
**Round 2**				
**Outcome (n=13)**	**Rating by 53 participants, n (%)**			
	**Critical**	**Important but not critical**	**Not important**	**Unable to score**
**Medication adherence across multiple medications**	**53 (100.0)***	0 (0.0)	0 (0.0)	0 (0.0)
Condition-specific outcomes	29 (54.7)	24 (45.3)	0 (0.0)	0 (0.0)
Mortality	15 (28.3)	35 (66.0)	3 (5.7)	0 (0.0)
**Treatment burden**	**51 (96.2)***	2 (3.8)	0 (0.0)	0 (0.0)
Falls	21 (39.6)	28 (52.8)	4 (7.5)	0 (0.0)
Frailty	24 (45.3)	27 (50.9)	2 (3.8)	0 (0.0)
**All adverse events and side effects**	**44 (83.0)***	9 (17.0)	0 (0.0)	0 (0.0)

Medication wastage	10 (18.9)	37 (69.8)	6 (11.3)	0 (0.0)
**Health-related quality of life**	**51 (96.2)***	1 (1.9)	1 (1.9)	0 (0.0)
Health-related quality of life for caregivers	18 (34.0)	32 (60.4)	3 (5.7)	0 (0.0)
Patient-carer satisfaction	39 (73.6)	10 (18.9)	2 (3.7)	2 (3.8)
**Healthcare utilisation**	**44 (83.0)***	9 (17.0)	0 (0.0)	0 (0.0)
**Cost-effectiveness**	**43 (81.1)***	8 (15.1)	2 (3.8)	0 (0.0)
**Round 3**				
**Outcome (n=7)**	**Rating by 50 participants, n (%)**			
	**Critical**	**Important but not critical**	**Not important**	**Unable to score**
Condition-specific outcomes	16 (32.0)	33 (66.0)	1 (2.0)	0 (0.0)
Mortality	7 (14.0)	39 (78.0)	4 (8.0)	0 (0.0)
Falls	11 (22.0)	35 (70.0)	4 (8.0)	0 (0.0)
Frailty	15 (30.0)	30 (60.0)	5 (10.0)	0 (0.0)
Medication wastage	7 (14.0)	35 (70.0)	7 (14.0)	1 (2.0)
Health-related quality of life for caregivers	10 (20.0)	36 (72.0)	4 (8.0)	0 (0.0)
**Patient-carer satisfaction**	**41 (82.0)***	7 (14.0)	1 (2.0)	1 (2.0)

*Figures in bold represent the outcomes that met the threshold for inclusion

Appendix 7. Participants' silent generation responses about the outcomes resulting from the nominal group consensus meetings

Participant No.	Professional background*	Silent generation responses
**Medication adherence across multiple medications**		
P1	P1 is a pharmacist, academic and a journal editor	*"It is important, but there are so many different ways to measure adherence that it is uncertain about the value of including it. There is currently no "gold standard" approach to measuring adherence across multiple medicines. Self report is a possibility, but if dispensed medicines were used it could limit uptake as there are additional hurdles and barriers to overcome to link dispensing data."*
P2	P2 is an academic	*"I do think it's important – if we want to 'improve' adherence we need try to measure it. It would be a critical outcome for an adherence intervention."*
P3	P3 is a pharmacist and an academic	*"Yes, absolutely! In my opinion, this is at the core of the definition of "adherence": if medicines are not taken as prescribed and the prescriber is not aware of this, wrong conclusions might be drawn and a therapy adapted based on erroneous background information. However, while I would absolutely want to see this indicator in the set, I still have many doubts in my study how to assess or measure adherence. I feel especially challenged measuring adherence in patients with cognitive impairment. In addition, I would want structure or numeric information (a score, for example), upon hospital admission, which is not yet done in "my" hospital at the moment. Therefore, an absolute YES to relevance, feasibility and measurability tentative."*
P4	P4 is an academic	*"Important to assess adherence to individual medicines because non-adherence to one particular medicines will adversely impact upon overall adherence. If issues are identified with individual medicines these can then be addressed and resolved thereby increasing overall adherence."*

Participant No.	Professional background*	Silent generation responses
P5	P5 is a nurse and an academic	*"This is probably a very important outcome and needs to be included definitely. If improvement of adherence is the target, adherence needs to be measured at different points of time. It is THE outcome for studies/trials seeking to improve adherence. Maybe there should be guidance what measurement to use to assess adherence."*
P6	P6 is an academic	*"Yes. This is an important measure for assessing interventions to improve adherence and is often not well reported, particularly in treatment RCTs. It can be difficult to measure e.g. using pharmacy claims, electronic devices, and self-reports, so use of validated or well established methods is essential."*
P7	P7 is a doctor, an academic and a journal editor	*"Yes. Important Key behavioral outcome."*
P8	P8 is a pharmacist and an academic	*"Yes; at least one but possibly two measure of medication adherence should be included, as this behavior is the proximal outcome to an intervention aim to improve medication adherence."*
P9	P9 is a pharmacist and an academic	*"YES- This should be included as it is central to clinical trials aimed at identifying interventions to improve adherence to appropriate polypharmacy."*
P10	P10 is a pharmacist and an academic	*"Yes. This is the key outcome measure. It is important to measure adherence to each medicine being taken because adherence may be different for different medicines."*
		**Treatment burden**
P1	P1 is a pharmacist, academic and a journal editor	*"It's important but the challenge of routinely collecting the outcome in a standardised approach limits it from a "should" include, even though it's important. Having too get linked data or self reported health care utilisation is onerous."*
P2	P2 is an academic	*"Yes very important. Polypharmacy poses significant burden on patients. If they are experience [sic] burden - be it physical, mental of financial, then they are likely to stop adhering to meds. Interventions that aim*

Participant No.	Professional background*	Silent generation responses
		to improve adherence to appropriate polypharmacy must target the elements of burden that are contributing to non-adherence. And if it isn't measured I don't think we can fully understand adherence."
P3	P3 is a pharmacist and an academic	*"Thank you for this relevant suggestion. I think treament [sic] burden perceived by the patient has a very relevant influence on adherence behaviour. In the beginning, I was wondering if we could use either "treatment burden" OR quality of life. But upon consideration, I would really keep both, following the hypothesis that a favourable outcome, like optimized quality of life, might contribute to patients' acceptance of a higher treatment burden. Again, if we keep it, standardizing assessment of treatment burden would be key in my opinion to have a valuable, comparable indicator."*
P4	P4 is an academic	*"Important to assess as overall treatment burden and the impact of an intervention in this regard. Non-adherence is likley [sic] to be related to several factors, not just the act of ingesting/using the medicine itself. Factors likely [sic] to impact adherence include coordination of healthcare appointments, repeat prescriptions requests, monitoring of conditions etc"*
P5	P5 is a nurse and an academic	*"Treatment burden influences adherence but I am not sure to what extend. Additionally, treatment burden might differ a lot between patients and disease states. It is also very difficult to measure. To my opinion, this core outcome needs to be discussed."*
P6	P6 is an academic	*"Yes, although it might depend on the intervention being evaluated. This seems quite a broad category - some of this is around utilisation of health care which would be useful for economic evaluations, while others are related to management including treatment and lifestyles. The latter might include self-management. This might need to be unpicked a bit more as to what treatment burden is."*
P7	P7 is a doctor, an academic and a journal editor	*"Important It is key to disentangle different dimensions in terms of functioning (physical, cognitive, social, affective...)"*

Participant No.	Professional background*	Silent generation responses
P8	P8 is a pharmacist and an academic	*"yes,[sic] but this outcome will likely serve as a moderator or modifier. I do not think it should be a primary outcome."*
P9	P9 is a pharmacist and an academic	*"YES. because [sic] in a number of cases this is overlooked and not given sufficient importance. The burden is usually studied in the more traditional side effects and possibly financial. However, it is exceptionally important to look at and measure the broader aspect of treatment burden which tends to be insufficiently documented."*
P10	P10 is a pharmacist and an academic	*"Yes. There amy [sic] be an element of cost to the patient, if the patient cannot afford or does not financially prioritise this indicator. This aspect is highly dependent on whether the patient is enetitled [sic] to tests and treatment for free or not. It will be important to clarify whether this refers only to drug treatment or also to other modalities."*
	**Health-related quality of life**	
P1	P1 is a pharmacist, academic and a journal editor	*"it's important but really more so for health economic evaluation. It is also unlikely to move with an intervention of this sort,"*
P2	P2 is an academic	*"Yes and no! I think that quality of life is a really important concept but the measures we have are so insensitive to change in these types of trials that it is meaningless. It creates burden on participants to have to fill in these questionnaires when they are somewhat meaningless. But then, we need them for most types of cost-effectiveness analysis and trials need to build this in."*
P3	P3 is a pharmacist and an academic	*"Absolutely include! If a therapy does not have a benefit, it will be much harder to motivate patients to be adherent. This is often reflected in patient bahviour [sic] towards therapies, which will need a long time period to see a benefit. See also comment on "therapeutic burden"."*

Participant No.	Professional background*	Silent generation responses
P4	P4 is an academic	*"Important to assess impact on QoL of any healthcare intervention however existing generic QoL instruments may not be sensitive enough to capture changes in QoL resulting from small increases/decreases in medication adherence."*
P5	P5 is a nurse and an academic	*"With this outcome I see the same problems as mentioned at treatment burden. Patients rate their QoL very different and many studies show no improvement of QoL if medication is changed or adjusted to patients` needs and it is very difficult to measure."*
P6	P6 is an academic	*"Yes. I think HRQoL is an important outcome in this area as it captures the individuals quality of life associated with the intervention in a patient reported outcome. It is a useful measures for economic evaluations where the cost-effectiveness can be evaluated in relation to gains in QoL. It is also a well recognised measure across different diseases and interventions so benchmarks for clinical improvements can be established."*
P7	P7 is a doctor, an academic and a journal editor	*"Important To be differentiated from point 2 [treatment burden] (where well-being is also measured)."*
P8	P8 is a pharmacist and an academic	*"no* [sic] *or mixed. there are too many intervening factors that impact this outcome. I look forward to the discussion about this outcome, as it is the most patient centered."*
P9	P9 is a pharmacist and an academic	*"YES. Prescribing 'appropriate' medication and assessing adherence without taking into consideration HRQL does not provide the full picture."*
P10	P10 is a pharmacist and an academic	*"No. This indicator is a nice to have. Patients will not see this as a basis to adherence to medicines."*
**All adverse events and side effects**		

Participant No.	Professional background*	Silent generation responses
P1	P1 is a pharmacist, academic and a journal editor	*"Absolutely 100% yes. This is critical to ensure safety and perceived safety."*
P2	P2 is an academic	*"Yes. I think we should measure this - adverse events are much like treatment burden, people will stop taking meds that cause them side effects. If we want to fully understand drug interactions, prescribing cascades etc in real patients then we should be collecting this data. We should also collect adverse withdrawal events if any of the interventions involve an element of deprescribing to improve adherence to appropriate prescribing."*
P3	P3 is a pharmacist and an academic	*"Yes. Based on the literature and personal experience, this experiences [sic] are often deal breakers for patient adherence and should be reflected. I only would like to point out that quality of life and adverse events are so tightly linked, that one might consider using "quality of life" as a surrogate parameter to reduce assessment burden."*
P4	P4 is an academic	*"Yes. I think it is important to assess all adverse events. I'm less certain about the value of assessing all side effects athough [sic] I appreciate that experience of side effects will impact upon adherence. I think assessment of all adverse events and side effects adds considerable complexity from a practical perspective so may mean that that this outcome is not well captured within trials."*
P5	P5 is a nurse and an academic	*"Yes, adverse events are a useful outome [sic] to measure and to improve adherence when considered. It makes an important difference for patients if they do not experience side-effects or adverse events are alleviated. Nevertheless, it is difficult when patients are on oral anti-tumor therapy as an example. Additionally, it is not always possible to connect one medication to one side-effect. But I would include this outcome in a core outcome set for improvement of adherence."*
P6	P6 is an academic	*"Yes – but possibly a secondary outcome in a trial. There is significant burden associated with polypharmacy so avoiding the adverse effects is crucial to avoid unnecessary harm caused by medicines.*

Participant No.	Professional background*	Silent generation responses
		This can sometimes result in hospitalisation which can lead to more serious outcomes and associated costs. The adverse effects are also related to the other outcomes such as quality of life etc.”
P7	P7 is a doctor, an academic and a journal editor	*“less [sic] important perhaps only major adverse events.”*
P8	P8 is a pharmacist and an academic	*“yes [sic], this is a proximal outcome to taking medications and is a necessary outcome. also [sic] a moderator of patient adherence in some/many cases.”*
P9	P9 is a pharmacist and an academic	*“Yes.. once again this is very important when considering adherence in a clinical trial. My main concern when documenting this type of info is: is the ADR or SE documented linked to the medication or not - Open for discussion- should there be a way to assess /ensure that what is documented is really linked to the medication- is it the pt perception that it is linked to the meds.”*
P10	P10 is a pharmacist and an academic	*“Yes But differnt [sic] types of adverse events will affect adherence differently. A patient will be OK with not taking a diuretic if this stops him from going out.”*
**Healthcare utilisation**		
P1	P1 is a pharmacist, academic and a journal editor	*“yes [sic], as before for treatment burden. This depends on what we will measure: pharmacy services, ambulance, paramedic, emergency department visits, hospitalisations,”*
P2	P2 is an academic	*“Yes and no! No because, in complex patients with multimorbidity and polypharmacy, there will unlikely be a lot of variation in healthcare utilisation over time anyway and unless there is a very long trial follow-up period, you won’t be able to pick up the down-stream implications of improving medication adherence and down-stream changes in utilisation. But then we need for cost-effectiveness...”*
P3	P3 is a pharmacist and an academic	*“yes [sic], I’m really happy with the Delphi outcome overall :-). I’m aware that this is a lot to measure. But if we want to consider the economic burden related to adherence issues, this is a multidimensional*

Participant No.	Professional background*	Silent generation responses
		parameter that I really like because it will offer information with a lot of room for interpretation. Again, this might also be reflected in treatment burden, quality of life and ADRs, which would allow for intersting [sic] regression models. However, where I'm a bit critical is the lacking factor of morbidity and/or therapy complexity which we might need to correct absolute numbers of health care utilization."
P4	P4 is an academic	*"Yes, I think it is important to capture healthcare usage as this forms an essential element of intervention cost-effctiveness [sic]"*
P5	P5 is a nurse and an academic	*"No, I would not include the outcome in the COS. Healthcare utilisation is influenced by so many factors, various in different countries, depending on health literacy, steady income and many more aspects. It is not suitable for a COS"*
P6	P6 is an academic	*"Yes - but depends on whether the interest is in an economic evaluation of the intervention in the trial. If economics is not included, then it may not be necessary to include this in the core outcomes. I would suggest it might not need to be a core outcome."*
P7	P7 is a doctor, an academic and a journal editor	*"less [sic] important as outcome"*
P8	P8 is a pharmacist and an academic	*"yes [sic], esp related to outcome 7 [cost-effectiveness]. However, this outcome may be 2-3 steps away for asymptomatic conditions but proximal re non-adherence for symptomatic conditions such as HF, COPD, pain"*
P9	P9 is a pharmacist and an academic	*"YES. As the degree and type of healthcare utilization may affect the degree of adherence."*
P10	P10 is a pharmacist and an academic	*"Yes. This links to the reply related to utilisation of resources and the financial burden. Will the healthcare services be at a cost or free? This will also depend on the accessibility of the services, e.g. waiting list and*

Participant No.	Professional background*	Silent generation responses
		queues to access the service. Also, accessibility in relation to transport and mobility to travel, support from relatives, transportation."
		**Cost-effectiveness**
P1	P1 is a pharmacist, academic and a journal editor	*"No. We need to ascertain safety and efficacy first rather than being driven by cost-effectiveness. It is important to consider it, but it is not a core outcome. If it were a core outcome, you would increase the cost of much research and limit idea testing and hypothesis generating research."*
P2	P2 is an academic	*"Yes. If we want to implement something, we need to know that it is effective, safe and cost-effective. That is what drives policy change so we must have this."*
P3	P3 is a pharmacist and an academic	*"I'm undecided. I'm not against using it - but I'm unsure what you want to measure here in the context of adherence. If an ADHERENCE measure worked or if a therapy the patient was prescribed is cost-effective? Therefore, I'm undecided."*
P4	P4 is an academic	*"Yes. Important to support decision making in terms commissioning and role [sic] out of successful interventions in routine practice"*
P5	P5 is a nurse and an academic	*"No. The measurability in different countries is questionable and there are probably very different aspects, and it needs to be discussed what is realy [sic] cost-effectiveness in consideration for example to qualtiy [sic] of live. I think it needs to be discussed. At this point I would not include cost-effectiveness."*
P6	P6 is an academic	*"No. Although I think this would be important to include and relates to some of the other outcome measures already mentioned e.g. healthcare utilisation, HRQoL, it might depend on the intervention being evaluated but costs should be included in trials to evaluate cost-effectiveness as a secondary outcome."*
P7	P7 is a doctor, an academic and a journal editor	*"No, less important for adherence"*

Participant No.	Professional background*	Silent generation responses
P8	P8 is a pharmacist and an academic	*"yes [sic]. Interventions can be costly, and we need to ensure the CE is permissible. the perspective and the specifics in measuring all aspects of a CEA can be done with planning."*
P9	P9 is a pharmacist and an academic	*"YES. Essential for implementation of any service targeted at enhancing adherence."*
P10	P10 is a pharmacist and an academic	*"No. Elderly patients will not go into such detail. They will see whether they can afford or not."*
		**Patient-carer satisfaction**
P1	P1 is a pharmacist, academic and a journal editor	*"Potentially no, satisfaction doesn't necessarily correlate. It is also important to note that this is not routinely collected data so may not be available if an intervention uses routinely collected data to measure the effect of an intervention by a health professional across an entire setting."*
P2	P2 is an academic	*"I am mixed on this one, but no. I think overall, very important to understand patient/carer experiences of health care but perhaps more qualitatively, something to explore in a process evaluation. I'm not sure as a quantitative measure it tells us much in an RCT. Certainly won't be an explanatory variable in analysis. Therefore, it is more burden on patients in terms of questionnaire completion etc with minimal value to the analysis."*
P3	P3 is a pharmacist and an academic	*"No. I'm not against it, because the dimension of this relationship is certainly influential on many other factors that have been chosen in the scope of the Delphi. However, I think we have to consider different sub-dimensions to do this topic justice: and that's medical competence v.s. social competence. Each might influence patient perception and therefore the relationship in different ways."*
P4	P4 is an academic	*"Important to capture patient perspectives of an intervention as process evaluation, but not necessary or primary"*
P5	P5 is a nurse and an academic	*"No. This is an interesting multidimensional measure and can be useful as an outcome to improve adherence. But one should take into account that these aspects influence each other like what was first*

Participant No.	Professional background*	Silent generation responses
		the hen or the egg and it is not primary. Are you adherent because you are satisfied with healthcare quality or because you are adherent and that improves healthcare quality."
P6	P6 is an academic	*"No, perhaps not in the core outcome set. I'm not sure it is a useful outcome to include in a trial of interventions to improve adherence to polypharmacy. Also, it will be a self-reported subjective measure which is less reliable as an outcome in a trial."*
P7	P7 is a doctor, an academic and a journal editor	*"No [sic] primary, important represents social support, control variable, almost a process evaluation"*
P8	P8 is a pharmacist and an academic	*"no [sic] or mixed, but mostly in terms of acceptability of an adherence intervention."*
P9	P9 is a pharmacist and an academic	*"Yes – Absolutely – I believe that this may have a significant impact on adherence in general but also on adherence to one drug as compared to another."*
P10	P10 is a pharmacist and an academic	*"No. Quality of services and interventions is not a direct determinant of adherence. The quality affects mainly aspects like communication."*

*Multiple professions are listed for some experts because they selected more than one job title.

ADRs: Adverse drug reactions; CE: Cost-effectiveness; CEA: Cost-effectiveness analysis; COPD: Chronic Obstructive Pulmonary Disease; HF: Heart failure; HRQL: health-related quality of life; QOL: Quality of life; RCTs: Randomised Controlled Trials; SE: Side effects.
